# Loop-Mediated Isothermal Amplification (LAMP) assay for the identification of *Echinococcus multilocularis* infections in canine definitive hosts

**DOI:** 10.1186/1756-3305-7-254

**Published:** 2014-05-30

**Authors:** Xingwei Ni, Donald P McManus, Hongbin Yan, Jifei Yang, Zhongzi Lou, Hongmin Li, Li Li, Mengtong Lei, Jinzhong Cai, Yanlei Fan, Chunhua Li, Quanyuan Liu, Wangui Shi, Xu Liu, Yadong Zheng, Baoquan Fu, Yurong Yang, Wanzhong Jia

**Affiliations:** 1 State Key Laboratory of Veterinary Etiological Biology/Key Laboratory of Veterinary Parasitology of Gansu Province/Key Laboratory of Zoonoses of Agriculture Ministry/Lanzhou Veterinary Research Institute, CAAS, Lanzhou 730046, P. R. China; 2 Molecular Parasitology Laboratory, Infectious Diseases Division, Queensland Institute of Medical Research, Brisbane QLD 4006, Australia; 3 Qinghai Academy of Animal Science and Veterinary Medicine, Xining 810016, P. R. China; 4 Gansu Provincial Center for Animal Disease Control and Prevention, Lanzhou Gansu Province 730046, P. R. China

## Abstract

**Background:**

Alveolar echinococcosis, caused by the metacestode larval stage of *Echinococcus multilocularis*, is a zoonosis of public health significance and is highly prevalent in northwest China. To effectively monitor its transmission, we developed a new rapid and cheap diagnostic assay, based on loop-mediated isothermal amplification (LAMP), to identify canine definitive hosts infected with *E. multilocularis*.

**Methods:**

The primers used in the LAMP assay were based on the mitochondrial *nad*5 gene of *E. multilocularis* and were designed using Primer Explorer V4 software. The developed LAMP assay was compared with a conventional PCR assay, using DNA extracted from the feces of dogs experimentally infected with *E. multilocularis*, on 189 dog fecal samples collected from three *E. multilocularis*-endemic regions in Qinghai province, the People’s Republic of China, and 30 negative control copro-samples from dogs from an area in Gansu province that had been subjected to an intensive de-worming program. Light microscopy was also used to examine the experimentally obtained and field collected dog copro-samples for the presence of *E. multilocularis* eggs.

**Results:**

The *E. multilocularis*-positivity rates obtained for the field-collected fecal samples were 16.4% and 5.3% by the LAMP and PCR assays, respectively, and all samples obtained from the control dogs were negative. The LAMP assay was able to detect *E. multilocularis* DNA in the feces of experimentally infected dogs at 12 days post-infection, whereas the PCR assay was positive on the 17th day and eggs were first detectable by light microscopy at day 44 post-challenge.

**Conclusion:**

The earlier specific detection of an *E. multilocularis* infection in dog copro-samples indicates that the LAMP assay we developed is a realistic alternative method for the field surveillance of canines in echinococcosis-endemic areas.

## Background

Alveolar echinococcosis (AE) is a parasitic zoonosis caused by the metacestode stage of *Echinococcus multilocularis*, the fox tapeworm. The disease is of significant public health relevance in the northern hemisphere, particularly in the northwestern parts of the Peoples’ Republic of China (PRC). Human AE has similar features to cancer [1] due to its infiltration of the liver and its invasive growth in this organ, together with its metastasis via the blood or lymphatic systems to other tissue sites [1]. Recent epidemiological studies of human AE have shown an increase in new cases in areas where *E. multilocularis* transmission had not been recorded previously [2]. The natural transmission cycle of *E. multilocularis* involves small mammal intermediate hosts which become infected after ingesting eggs released in the feces of infected foxes or other canines in a predator–prey relationship; in turn, canines become infected by ingesting tissues of infected small mammals. Transmission to humans occurs when eggs are ingested accidentally so that infected canine definitive hosts act as the source of disease transmission and human infection [3,4].

Prior to the development of PCR-based methods, the most reliable procedure for the diagnosis of *Echinococcus* spp. in definitive hosts, especially in developing countries, was necropsy; using this approach, worm burdens can be estimated and parasites collected for identification [5]. However, necropsy usually results in biased sampling as, generally, only stray or unwanted dogs are necropsied. Despite the early promise shown by the use of copro-antigen detection of *E. multilocularis* by capture-ELISA [6], the test shows low specificity compared with necropsy for diagnosis and surveillance purposes. There is a high rate of cross-reactivity with other helminth infections [7], particularly *E. granulosus*[6], and sensitivity is also highly dependent on worm burden [5]. Copro-DNA-based tests can provide an alternative method of diagnosis [8] and since the first PCR-based method described by Bretagne *et al*. [9] for the detection of *E. multilocularis* DNA in the feces of foxes, the technique has been improved. A conventional PCR-based test using the mitochondrial (mt) *12S rRNA* gene [10,11], a multiplex-PCR test [12] and a nested PCR assay [10] have been shown to be of diagnostic value for the detection of *E. multilocularis* infections in canines. However, for routine laboratory diagnosis and surveillance, these methods have a considerable drawback, since the sensitivity of PCR can be severely affected by inhibitory factors present in fecal samples [10]. Furthermore, the expensive facilities and reagents and the relatively long time required for test completion are additional disadvantages [13]. Consequently, a more rapid, sensitive and specific diagnostic method for the identification of *E. multilocularis* infections in canines would be of considerable value. A loop-mediated isothermal amplification (LAMP) method, developed by Notomi *et al.*[14,15], has been shown to rapidly detect and differentiate pathogen species and has higher specificity and sensitivity than conventional PCRs for detecting DNA in fecal samples [16,17]. A LAMP assay has recently been described for *E. granulosus* copro-detection [13] but it has not been tested on canine stool samples collected in the field. Moreover, to date, no such test is available for the identification of *E. multilocularis* infections in canines. With the increasing numbers of mitochondrial (mt) DNA gene sequences becoming available, mt genes are being increasingly applied in species identification, molecular taxonomy, evolutionary studies and diagnosis, and in molecular epidemiological investigations of the parasitic helminths [18,19]. Recently full-length mt DNAs have been sequenced in our laboratory and in others for a large number of cestode species parasitic in the small intestine of carnivores [18-22]. The *nad*5 gene is a protein-encoding gene with substantial nucleotide variability, which makes it highly suitable for designing LAMP primers for the identification of related species [18]. At least five and up to nine species are accepted within the genus *Echinococcus*[21-23]; three species - *E. multilocularis*, *E. granulosus* and *E. shiquicus* – occur sympatrically on the Qinghai-Tibet Plateau of China [24,25]. The present study describes the establishment of a LAMP assay for the detection of *E. multilocularis* DNA in dog feces and is based on a fragment of the *nad*5 gene of *E. multilocularis.* We have evaluated its applicability for testing dog fecal samples collected during routine *E. multilocularis* surveillance in China and compared its practical value with conventional microscopy and a traditional PCR-based assay.

## Methods

### Ethical statement

All experiments using mice and dogs were undertaken under strict Chinese experimental animal clearances and animals at all times were treated in accordance with animal ethics procedures and guidelines for animal husbandry of the Institutional Ethics Committee of Lanzhou Veterinary Research Institute, Chinese Academy of Agricultural Sciences. The study and the use of animals were approved by this Committee, (Approval No. LVRIAEC2010-005).

### *E. multilocularis* material

A larval isolate of *E. multilocularis* used in all experiments was obtained originally in 2010 from a naturally infected plateau pika (*Ochotona curzoniae*) in Shiqu county, Sichuan province, P.R. China, and was passaged routinely by intraperitoneal passage in white mice. Protoscoleces were obtained by macerating the multilocular cystic masses collected from the peritoneal cavity of the infected mice and were checked by microscopy to determine viability. Only parasites having over 95% viability were used either to experimentally infect dogs or were stored immediately at −70°C for DNA isolation.

### Experimental infection of dogs

Common breed (crossbred) puppies were born from two pregnant dogs, purchased at a local market, at Lanzhou Veterinary Research Institute. The dogs were treated with albendazole (5 mg/kg body weight on three consecutive days) in order to remove cestodes and nematodes from their intestines two months prior to study commencement, and the dogs were certified helminth-free by routine microscopic examination of feces. The animals were kept in the experimental facility at Lanzhou Veterinary Research Institute for two weeks prior to commencement of the study to allow them to adapt to the living conditions and diet. All animals were fed a heat-treated meal once daily and examined weekly by a veterinarian. Six dogs (kept in individual cages; circa six months old; average weight 10 kg) each received orally single inoculation of approximately 10,000 *E. multilocularis* protoscoleces, administered in saline as part of their normal meal. Fecal samples were collected daily from the bottom of the individual cages, placed into sterilized 50-ml containers with tight fitting lids, and stored at −70°C until use. The six dogs were sacrificed humanely to determine their *E. multilocularis* infections at day 50 post-challenge. Their intestines were removed, the gut contents were sedimented repeatedly with physiological saline and adult worms were manually picked with needles or glass straws.

### Collection of dog feces in the field

Fecal samples (n = 189) were collected from individual dogs in three *E. multilocularis-*endemic areas of Qinghai province, P.R. China. All collected fecal samples were stored at −70°C before examination by microscopy and for DNA isolation. A further 30 fecal samples from unwanted domestic dogs were collected as negative-controls from an area of Gansu province, where mass dog treatment with praziquantel (10 mg/kg) had been carried out monthly for more than one and a half years, and where no human AE cases had been recently recorded. The 30 dogs were sacrificed humanely to confirm whether they were cestode-free. In brief, after the whole intestinal contents of each dog were collected, a small amount was kept at −70°C until use and the remainder was examined to determine whether any *Echinococcus* worms were present.

### Microscopic examination of fecal samples for the presence of taeniid eggs

All dog fecal samples were subjected to a conventional saturated sodium chloride (NaCl) flotation method for isolation of eggs [26]. Briefly, 2 g feces were washed with distilled water and then sedimented by centrifugation at 2,500 × g for 10 min with the supernatant being discarded. Then the sediment was suspended in saturated NaCl solution and any eggs present were observed by light microscopy.

### Parasite and host DNA samples

Genomic DNA from *E. multilocularis* protoscolex tissue, obtained from infected mice (t-g-DNA), and genomic DNA (f-g-DNA), isolated from fecal samples obtained from the experimentally infected dogs, were extracted using Axyprep™ Multisource Genomic DNA Miniprep Kits (Axygen, CA, USA) and QIAamp DNA Stool Mini Kits (Qiagen, Germany), respectively. The t-g-DNA samples were used as positive controls for establishment of the sensitivity of the LAMP assay. Genomic DNA samples (g-DNAs) from *E. granulosus* (common sheep strain; G1 genotype), *E. shiquicus*, *T. hydatigena*, *T. pisiformis*, *T. taeniaeformi*s, *T. multiceps* and *Dipylidium caninum* were used to determine the specificity of the *E. multilocularis* LAMP assay. Apart from the *T. taeniaeformis* DNA, which was kindly provided by Viktor Dyachenko, Institute for Infectious Diseases and Zoonoses, Ludwig-Maximilians-University of Munich, Munich, Germany and the *E. shiquicus* DNA, which was extracted from a cyst collected from a naturally infected plateau pika in Shiqu in 2011, all the other cestode DNA samples were obtained from experimentally infected dogs at Lanzhou Veterinary Research Institute. In addition, intestinal contents (200 mg) and fecal samples (200 mg) (n-f-DNA) from uninfected dogs were obtained from newly born pups and the DNAs were extracted to serve as negative controls. The concentrations of the DNA samples were measured using a Nanodrop 2000 spectrophotometer (Thermo Scientific, China).

### Conventional PCR assay

Conventional PCR was carried out for comparative purposes. The PCR primers EMH17/EMH15 were used to amplify a 200 bp fragment of the mt *12S rRNA* gene of *E. multilocularis* (GenBank accession No. AB031351) [8,10]. The PCR amplification reactions took place in a total volume of 50 μl consisting of 10 mM Tris–HCl (pH 9), 50 mM KC1, 2 mM MgCl_2_, 200 μM of each dNTP, 0.2 μM each primer, 1.25 U *Taq* polymerase (TaKaRa, Dalian, China). The thermal cycling conditions used were as follows: 95°C for 4 min, 35 cycles at 94°C for 30 sec, 53°C for 30 sec, and 72°C for 30 sec, with a final extension at 72°C for 10 min. PCR products were visualized on a 1.5% (w/v) agarose gel with ethidium bromide.

### LAMP assay

LAMP primers were designed based on the mt *nad*5 gene (GenBank accession No. AB031351) [20] using Primer Explorer V4 software (http://primerexplorer.jp/elamp4.0.0/index.html). The forward inner primer (FIP), backward inner primer (BIP), and two outer primers (F3 and B3) were specifically designed to recognize six separate regions within the *nad*5 gene. The *nad*5 nucleotide sequences for other canine tapeworms were checked or involved in the design of primers [18-22]. Primers were validated using BLAST software (http://www.ncbi.nlm.nih.gov/BLAST). All the primer sequences are listed in Table 1 and alignment of target *nad*5 nucleotide sequences for *Echinococcus* species was performed with the primers (Additional file 1: Figure S1). An *Eco*RI restriction site (*gaattc*) was introduced into the FIP and BIP primers for restriction enzyme digestion analysis of the LAMP products.

**Table 1:** **Nucleotide sequences of the LAMP primers (licensed patent no. ZL201110346474.8) targeting the mt *nad*** **5 gene**

**Primer name**	**Sequence (5′ → 3′)**
FIP	TTAACCAACCAATAACAACCCAGT*gaattc*GTGGTGTTAGTTATTTGGTTAGG
BIP	ATGTGACGTTTGGTGTGGTAGTTA*gaattc*AAGAACCACCAAAATAATGTCT
F3	GTGTGTTGCTATATTGCTTGT
B3	AACTTTAACAACATACACCTAGT

Note: The lower case italicized *gaattc* in the primers FIP and BIP shows the position of the introduced *Eco*RI restriction site.

The LAMP reaction was performed in a 25 μl volume with 2 μl of *E. multilocularis* g-DNA, 1.8 μl of primer mix (40 pmol each of FIP and BIP, 5 pmol each of F3 and B3), 1.0 μl of *Bst* DNA polymerase (8 U), 12.5 μl of reaction buffer (1.6 M betaine, 40 mM Tris–HCl [pH8.8], 20 mM KCl, 20 mM (NH_4_)_2_SO_4_, 16 mM MgSO_4_, 0.2% (v/v) Tween 20 and 2.8 mM dNTPs) and 7.7 μl of ddH_2_O. LAMP products were analyzed on a 1.5% (w/v) agarose gel with ethidium bromide, and the LAMP products were visually determined after the addition of a 1/10 dilution of SYBR Green I (Invitrogen) to the reaction tube as well.

In order to determine the optimal reaction temperature and time, the reaction mixture was incubated at 60°C, 61°C, 62°C, 63°C, 64°C and 65°C, respectively, for 30 min and then heated at 80°C for 5 min to terminate the reaction; then six different reaction time periods (10, 20, 30, 40, 50 and 60 min) were compared at the optimal reaction temperature.

### The specificity and sensitivity of the LAMP assay

To verify the specificity of the LAMP assay for detection of *E. multilocularis* DNA, the LAMP primers were tested using g-DNAs from *E. granulosus* (G1 genotype), *E. shiquicus*, *T. hydatigena*, *T. pisiformis*, *T. taeniaeformis*, *T. multiceps*, *D. caninum*, and n-f-DNA (fecal samples from cestode-free dogs) and dog intestinal tissue as negative controls. To further confirm the specificity of the LAMP amplification, the sequences of the LAMP amplicons were determined using a modification of the method described by Nkouawa *et al*. [27]. Briefly, the *Eco*RI-digested LAMP products were cloned into pMD-18 T vectors (TakaRa, Dalian, China), and recombinant plasmids were sequenced by Sangon Biotech Co., Ltd. (Shanghai, China). In order to determine the sensitivity of the LAMP assay, *E. multilocularis* t-g-DNA was diluted to 10 ng/μl and then successively diluted 10 times by the addition of 1 μl of a 1/10 dilution of the previous concentration. The same dilution procedure was also performed on DNA samples from dog-feces (f-g-DNA) obtained at different days post- experimental infection. In addition, the minimum number of eggs detected by the LAMP assay was determined in the experiments with feces spiked with eggs obtained from *E. multilocularis* adults collected from one of the experimentally infected dogs. The eggs were counted, mixed with feces from an uninfected dog and the feces were then frozen until use. These experiments were done in triplicate.

### Examination of field obtained fecal samples

The 189 field-obtained copro-samples (collected from the *E. multilocularis-*endemic areas) were examined by the LAMP and PCR assays. The presence of taeniid eggs was confirmed by microscopy. The f-g-DNA extracted from the feces of an experimentally infected dog was used as positive control. The LAMP and PCR products were electrophoresed on a 1.5% (w/v) agarose gel containing ethidium bromide and photographed using a gel documentation system.

### Statistical analysis

Differences in sensitivity between the LAMP test, PCR assay and microscopy were analyzed using a One-Way ANOVA with *post hoc* LSD tests and the Chi-square test using SPSS 11.5 [28].

## Results

### The optimal reaction temperature and time for the LAMP assay

The optimal reaction temperature and time for the LAMP assay proved to be 63°C and 40 min, respectively.

### Analysis of the digested LAMP products

**Figure 1:**
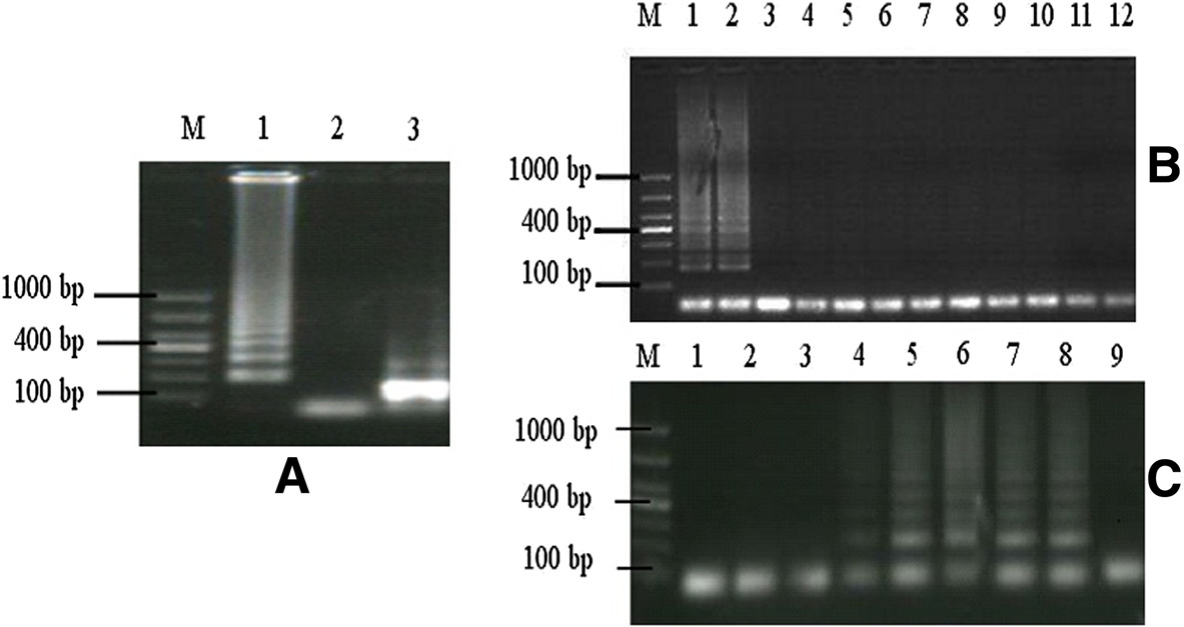
**Establishment of the LAMP assay.** (**A**: Left Panel) Amplification of LAMP of f-DNA (63°C for 40 min) and restriction digestion of LAMP products. Lanes: M, DNA marker; 1, LAMP products of f-DNA; 2, water control; 3, *Eco*RI digestion of LAMP products. (**B**: Right-top Panel) Specificity of LAMP assay for *E. multilocularis* DNA. Lanes: M, DNA marker; 1 and 2, g-DNA from *E. multilocularis* larvae and f-DNA; 3 to 9, g-DNA of *E. granulosus* (G1 strain), *E. shiquicus*, *T hydatigena*, *T. pisiformis*, *T. taeniaeformis*, *T. multiceps*, *D. caninum* parasites; 10, g-DNA from dog intestinal tissues; 11, no f-DNA; 12, water control. (**C**: Right-bottom Panel) Sensitivity of LAMP method using different numbers of *E. multilocularis* eggs per gram of feces. Lanes: M, DNA marker; 1 to 5, one to five eggs; 6, ten eggs; 7, fifteen eggs; 8, twenty eggs; 9, negative control.

The LAMP products demonstrated typical patterns of ladder-like bands on agarose gels and their *Eco*RI digestion products were as expected (Figure 1A). Also all 16 randomly chosen LAMP amplicons were confirmed to contain the correct target gene sequence.

### Experimentally infected dogs

Three of the six dogs yielded 415, 353 and 209 *E. multilocularis* worms; no worms were recovered from the other three dogs.

### Specificity and sensitivity of LAMP in experimental samples

*E. multilocularis* t-g-DNA and f-g-DNA and g-DNAs extracted from the other parasites and dog host intestinal tissues were tested to determine the specificity of the LAMP assay for *E. multilocularis* DNA. Only the target gene fragments in *E. multilocularis* t-g-DNA and f-g-DNA produced amplified products (Figure 1B). The PCR and LAMP assays showed a similar level of sensitivity (detecting as low as 1 pg DNA) when using different concentrations of *E. multilocularis* t-g-DNA (data not shown). However, a positive result was obtained on day 12 post infection in the fecal samples of three infected dogs using the LAMP assay, whereas a positive result was not obtained until day 17 post infection using the traditional PCR test, thereby indicating the former was more sensitive with a statistically significant difference (p < 0.05) between the two assays. The LAMP and PCR assays were substantially more sensitive than microscopy, as eggs were not detected visually in feces until day 44 post challenge (p < 0.01) (Table 2). Fecal samples from three of the experimentally infected dogs were shown to be continuously positive by LAMP, PCR, and light microscopy until the dogs were sacrificed. The limit of detection of the LAMP assay was four *E. multilocularis* eggs per gram of feces determined three times (Figure 1C).

**Table 2:** **The earliest time when fecal samples of *E. multilocularis*-experimentally infected dogs tested positive with the LAMP and PCR assays and by microscopy**

**Method**	**Earliest day for positivity**			**Mean (day)**	*P value
	**Dog 1**	**Dog 2**	**Dog 3**		
LAMP	12	12	13	12.3	0.03^a^
PCR	17	17	18	17.3	<0.001^b^
Microscopy	42	44	46	44	<0.001^c^

Note: a, LAMP versus PCR; b, LAMP versus microscopy; c, PCR versus microscopy.

*Statistical analysis used one-way ANOVA with *post hoc* LSD tests: P < 0.05 meaning statistical significance; P < 0.01 indicating significant difference; P < 0.001 showing extreme significant difference.

### Performance of the LAMP assay using dog fecal samples collected in the field

The highest positivity rate (31/189; 16.4%) was achieved using the LAMP assay with dog feces collected from *E. multilocularis*-endemic areas [29] (Table 3). All 30 negative fecal control samples were negative by LAMP, PCR and microscopy. Sixteen of the positive LAMP products were randomly selected for analysis by DNA sequencing, which confirmed their identity. Twenty one fecal samples that were *E. multilocularis* LAMP-positive but PCR-negative were shown to be negative again when subjected to a second round of the PCR assay. The outcomes of the field collected fecal samples using the LAMP and PCR assays and microscopy are summarized in Table 3. The positivity rate for the PCR assay and microscopy (10/189; 5.3%) was the same but some samples positive using one method were negative by the other and vice versa. The LAMP positive samples included all the positive samples determined by both the PCR assay and microscopy and some other samples that were negative by these two methods. Therefore, the LAMP assay was significantly more sensitive (P < 0.001; Pearson chi-square test) than either of the other two methods used.

**Table 3:** Number of field collected dog fecal samples shown to be positive or negative by the LAMP assay, PCR method and microscopy

**Number of samples**	**Assay outcomes**		
	**LAMP**	**PCR**	**Microscopy**
5	Positive	Positive	Positive
5	Positive	Positive	Negative
5	Positive	Negative	Positive
16	Positive	Negative	Negative
158	Negative	Negative	Negative
Total 189	*31 positive samples	10 positive samples	10 positive samples
	158 negative samples	179 negative samples	179 negative samples

*The LAMP assay exhibited a significantly higher level of sensitivity than either the PCR method or microscopy (P < 0.001; Pearson chi-square test).

## Discussion

LAMP is a novel nucleic acid amplification method, involving the use of four DNA primers, which has been developed as a useful tool for the epidemiological surveillance of several parasitic infections [16,17], and it has potential value for the specific and sensitive identification of adult tapeworm infections in dogs and other canines. The LAMP primers used in this study amplified the *nad*5 target gene from the g-DNA of *E. multilocularis*, but not from the g-DNAs of any other cestode tested, including the closely related *E. granulosus* and *E. shiquicus.* Therefore, the LAMP assay exhibited high specificity for application in the diagnosis of *E. multilocularis* infection in canine hosts similar to that recently reported for the differential detection of *Taenia* species from humans using fecal specimens [27]. In addition, no nucleotide variation was observed in the primer regions or sites of 30 *E. multilocularis* isolates collected from field mice in China (data not shown), which make the primers very effective. The assay can provide a very useful tool for differential diagnosis between co-endemic *E. granulosus*[13] and *E. multilocularis* in canines, thereby providing an improved surveillance method for discriminating the two species in order to provide the accurate information required for the implementation of echinococcosis control programs. Whereas the PCR and LAMP methods employed in this study exhibited similar levels of sensitivity when tested with different concentrations of *E. multilocularis* g-DNA, the latter displayed a higher sensitivity in the detection of f-DNA from dogs experimentally infected with *E. multilocularis*, disclosing an infection in challenged dogs about a week earlier. A possible explanation for this may be the presence of inhibitors in the f-DNA templates, which can result in lower sensitivity and reproducibility of PCR assays [27,30], so that more eggs or parasite DNA may be required to obtain a positive reaction. The *Taq* DNA polymerase used in PCR is more often inactivated and affected by these inhibitors than the *Bst* DNA polymerase used in LAMP [27]. It is noteworthy that other studies have shown the LAMP method is also more sensitive in detecting other pathogens in fecal samples [27,31]. Nevertheless, an internal control to the PCR needs to be included to check for significant inhibition in the future. Alternatively spiked fecal samples with *E. multilocularis* t-g-DNA can be used to demonstrate possible inhibition, which are used to assess the sensitivity of PCR and LAMP.

The results of this study indicate that the LAMP method is much more sensitive than both conventional PCR and light microscopy for the identification of *E. multilocularis* in dog fecal samples collected in the field. Furthermore, the LAMP amplification can take place at an isothermal temperature in a water bath or a heating block, and it requires one reaction of 40 min compared with PCR which generally requires two hours, or more, involving denaturation, annealing and extension reaction steps. Overall, therefore, the LAMP assay is simpler and faster than the PCR method and is an approach that has been applied successfully for the detection of a range of viral, bacterial, fungal, and parasitic infections [17,32,33].

Therefore, considering it is less expensive and more rapid than traditional PCR methods, LAMP is an attractive, alternative diagnostic tool for use in resource-poor countries, where parasites are prevalent and facilities are relatively undeveloped [34]. Furthermore, if the amplified mix is combined with SYBR Green [16,35] or another dye such as hydroxynaphthol blue [36], the test can be immediately visualized to distinguish a positive LAMP reaction from a negative control, thereby providing ease of use in the field.

## Conclusion

In summary, the LAMP method we have developed has significant potential for the diagnosis of *E. multilocularis* infected canines in *Echinococcus*-endemic regions, particularly in underdeveloped countries such as China. It is an important new advance for early diagnosis and is a potentially useful epidemiological surveillance tool since it provides an accurate, sensitive, affordable, and easy-to-use method and a practical alternative to PCR for the routine diagnosis of *E. multilocularis* infections in dogs, foxes and other canines.

## Competing interests

The authors declared that they have no competing interests.

## Authors’ contributions

Conceived and designed the experiments: XWN DPM HBY JFY ZZL YRY WZJ. Performed the experiments: XWN HBY JFY ZZL HML WZJ. Analyzed the data: XWN DPM HBY YRY WZJ. Contributed reagents/materials/analysis tools: XWN HBY ZZL HML LL MTL JZC YLF YDZ BQF YRY WZJ. Wrote the paper: XWN DPM YRY WZJ. All authors read and approved the final version of the manuscript.

## Supplementary Material

Additional file 1: Figure S1 Multiple sequence alignment of mt *nad*5 sequences of canine *Echinococcus* spp. using Clustal 2.1. Eca-G7: *E. canadensis* (*E.g.* G7), Eca-G6: *E. canadensis* (*E.g.* G6), Eca-G10: *E. canadensis* (*E.g.* G10), Eca-G8: *E. canadensis* (*E.g.* G8), Eor-G5: *E. ortleppi* (*E.g.* G5), Ee-G4: *E. equinus* (*E.g.* G4), Eg: *E. granulosus*, Ef: *E. felidis*, Es: *E. shiquicus*, Ev: *E. vogeli*, Eo: *E. oligarthrus*, Em: *E. multilocularis*. Click here for file
