# Supplementary material for: Loop-Mediated Isothermal Amplification (LAMP) assay for the identification of Echinococcus multilocularis infections in canine definitive hosts
Source: Parasit Vectors. 2014 May 30;7:254. doi: 10.1186/1756-3305-7-254 (PMC4081488; doi:10.1186/1756-3305-7-254)

**Additional file 1: Figure S1.** Multiple sequence alignment of mt *nad*5 sequences of canine *Echinococcus* spp. using Clustal 2.1. Eca-G7: *E. canadensis* (*E.g.* G7), Eca-G6: *E. canadensis* (*E.g.* G6), Eca-G10: *E. canadensis* (*E.g.* G10), Eca-G8: *E. canadensis* (*E.g.* G8), Eor-G5: *E.* *ortleppi* (*E.g.* G5), Ee-G4: *E. equinus* (*E.g.* G4), Eg: *E. granulosus*, Ef: *E. felidis*, Es: *E. shiquicus*, Ev: *E. vogeli*, Eo: *E. oligarthrus*, Em: *E. multilocularis*.


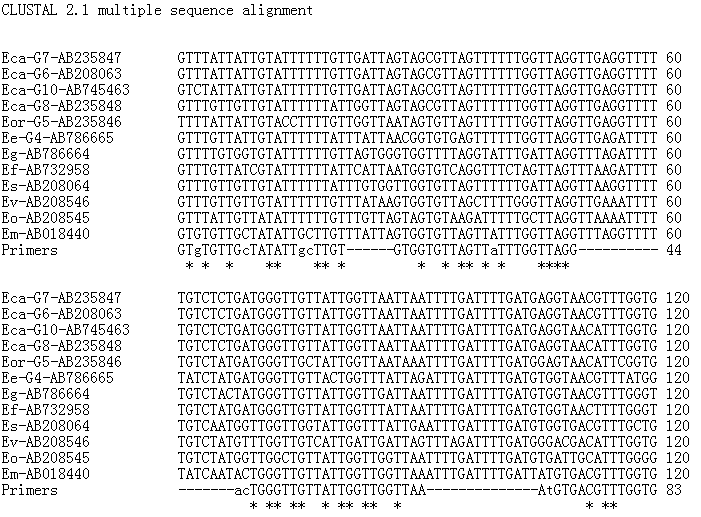


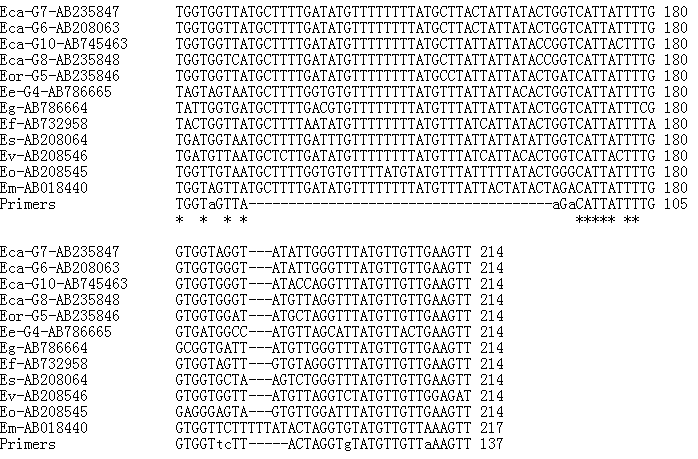

Supplement: Additional file 1: Figure S1 — Multiple sequence alignment of mt nad5 sequences of canine Echinococcus spp. using Clustal 2.1. Eca-G7: E. canadensis (E.g. G7), Eca-G6: E. canadensis (E.g. G6), Eca-G10: E. canadensis (E.g. G10), Eca-G8: E. canadensis (E.g. G8), Eor-G5: E. ortleppi (E.g. G5), Ee-G4: E. equinus (E.g. G4), Eg: E. granulosus, Ef: E. felidis, Es: E. shiquicus, Ev: E. vogeli, Eo: E. oligarthrus, Em: E. multilocularis. [file 1756-3305-7-254-S1.doc]
